# Adoption of Personal Health Records in Dutch Hospitals and Private Medical Clinics: Longitudinal Study

**DOI:** 10.2196/71915

**Published:** 2025-08-11

**Authors:** Doris van der Smissen, Christine Leenen-Brinkhuis, Kim M E Janssens, Petra J Porte, Marcel A L M van Assen, Anne Marie Weggelaar-Jansen

**Affiliations:** 1 Tranzo, Scientific Center for Care and Wellbeing, Tilburg School of Social and Behavioral Sciences, Tilburg University, Postbus 90153, Tilburg, 5000 LE, The Netherlands, 31 31134662969; 2 Dutch Hospital Association, Utrecht, The Netherlands; 3 Digizo.nu, Zeist, The Netherlands; 4 Erasmus School of Health Policy and Management, Erasmus University Rotterdam, Rotterdam, The Netherlands; 5 Department of Methodology and Statistics, Tilburg School of Social and Behavioral Sciences, Tilburg University, Tilburg, The Netherlands; 6 Department of Sociology, Utrecht University, Utrecht, The Netherlands; 7 Clinical Informatics, Eindhoven University of Technology, Eindhoven, The Netherlands

**Keywords:** personal health records, patient portals, adoption, adoption processes, eHealth, Technology Adoption and Readiness Scale, TARS, longitudinal study

## Abstract

**Background:**

Personal health records (PHRs) allow patients to monitor, track, and manage their health, improve patient-provider communication, and enable the collection, management, and use of health data from various health care organizations. Despite their potential to empower patients and facilitate health care professionals’ practices, PHR adoption remains challenging.

**Objective:**

This study aims to examine longitudinal PHR adoption in Dutch general hospitals, academic hospitals, and private medical clinics.

**Methods:**

We studied PHR adoption using the Technology Adoption and Readiness Scale (TARS) over time. TARS evaluates eHealth adoption, implementation, and embedment in organizations. It includes 7 subscales: coherence (sense-making and understanding of the new practice), cognitive participation (engagement with the new practice), contextual integration (integrating the new practice in the organization’s overall goals and structure), skill set workability (integrating with existing working practices and skills), relational integration (the fit with existing professional relationships), interactional workability (whether the practice enables interactions), and reflexive monitoring (appraisal of the new practice). The TARS consists of 32 statements using a 6-point Likert scale ranging from completely disagree to completely agree. After each TARS statement, participants had the opportunity to explain their answer (optional open question). Contact persons per organization of a national program completed the questionnaire in the autumn of 2021 (n=143), 2022 (n=131), and 2023 (n=101). Mixed-model analysis using IBM SPSS was done for the quantitative data and content analysis was done for the qualitative data.

**Results:**

Significant improvements in coherence (*P*<.001), cognitive participation (*P*=.01), and skill set workability (*P*=.02) were observed over time. Conversely, interactional workability showed a significant decline (*P*=.01). No changes were observed for relational integration (*P*=.06) and reflexive monitoring (*P*=.77). The development of contextual integration differed over time between the different types of organizations (*P*<.001); in private medical clinics, contextual integration decreased, while it increased in both general and academic hospitals. General hospitals consistently scored lower on coherence, cognitive participation, and reflexive monitoring compared to private medical clinics. Qualitative analysis indicated that communication about PHRs improved sense-making and increased professional engagement and commitment. Barriers to adoption included technical issues, financial constraints, concerns about the digital skills of patients and professionals, and privacy and data security concerns.

**Conclusions:**

From 2021 to 2023, PHR adoption improved in general hospitals, academic hospitals, and private medical clinics in the Netherlands within several domains of PHR adoption. However, several barriers remained, including technical barriers, financial constraints, and privacy and security concerns. To overcome these barriers, the technical integration of available data from the electronic health record within the PHR should be improved and privacy-related issues should be resolved. Consistent communication about the potential of PHRs is required to increase the awareness that will enhance both PHR engagement and adoption by the target groups, patients, and health care professionals.

## Introduction

Personal health records (PHRs) offer benefits to patients, including greater knowledge about their disease, increased engagement in their care, improved treatment adherence, enhanced self-management, and better clinical outcomes [1]. PHRs are designed for patients to monitor, track, and manage their health by collecting, managing, and using their health data and simultaneously are intended to improve patient-provider communication [2]. Despite their potential to empower patients and facilitate health care professional practices, PHR adoption remains challenging. However, the specific barriers and strategies to overcome these are not yet well understood [3].

In the literature, various definitions of PHRs overlap and are used interchangeably with the definitions of the patient portal (PP) and personal health environment (PHE). Consequently, definitions often lack coherence in the current literature [4-7]. The unclear distinction between PHRs, PPs, and PHEs complicates any comparisons. Therefore, we first elaborate on their similarities and differences to clarify the definitions. Generally, there are 3 key differences in the definitions of PPs and PHRs. First, PPs are owned, managed, and controlled by health care organizations or providers [8], whereas PHRs are owned, managed, and controlled by patients [9,10]. Consequently, PHRs can include information recorded by patients, which is not included in electronic health records (EHRs) or in PPs [9]. Second, PPs typically contain health-related information from a single healthcare provider or organization, while PHRs can aggregate information from multiple health care entities and various eHealth technologies [9,10]. Third, data in PPs are automatically updated from the EHR, whereas patients can also update their PHR data [11]. Thus, a PHE combines the electronic and personal health records (EHR + PHR) in a single portal allowing patients to access their medical data from various health care organizations, record their own medical data, and control access to their medical data. This description of a PHE largely overlaps with the definition of PHR. As the term “PHE” is rarely used in the literature [6,7], we use the term PHR in our study, defined as: PHRs support the collection, management, and use of health data from various health care organizations and patients themselves, and empower patients to monitor, track, and stay informed about their health [2]. PHR functionalities are described as: give patients insights into their recent doctor visits, test results, discharge summaries, medications, immunizations, allergies, administrative support in secure messaging between providers and patients, book and check appointments, and request prescription refills [9,10,12,13].

Although many studies examine the functionality, accessibility, and usability of PHRs, as well as their barriers and benefits [5,9,10,12], fewer studies focus on PHR adoption by patients and health care professionals [14], which is essential to ensure PHR usage. Rouleau et al [15] mapped the theories, models, and frameworks in a scoping review that addressed the adoption, implementation, and embedment of eHealth. They identified a total of 68 theories, models, and frameworks, of which only 6 were consistently applied in the literature, namely the Normalization Process Theory (NPT), the Consolidated Framework for Implementation Research, the Reach, Effectiveness, Adoption, Implementation, and Maintenance Framework, the Technology of Acceptance Model, the Unified Theory on Acceptance and Use of Technology, and the Diffusion of Innovation Theory [15]. The more recently developed Non-Adoption, Abandonment, Scale-Up, Spread, and Sustainability framework also evaluates eHealth implementation [16]. Given their similarities, the selection of any one theory or framework to evaluate an eHealth tool depends on the research aims, needs, and local context [15]. For our study, the NPT framework was considered most suitable to apply as it addresses the implementation, embedment, and adoption processes in relation to social contexts that are understudied in relation to PHRs.

Many studies and multiple systematic reviews apply the NPT on different topics [15]. For instance, the review by May et al [17] shows that the NPT was effective, useful, and applicable to assess whether eHealth interventions were successfully implementable. The NPT was helpful during the development and evaluation of dynamic implementation processes [17] and randomized controlled trials [18]. At the start of this study, the NPT was the only framework accompanied by validated questionnaire: the Technology Adoption and Readiness Scale (TARS) [19-21]. The TARS is based on the theoretical concepts of the NPT, enabling evaluation of eHealth adoption in organizations [19-21]. The TARS can be used to assess processes essential to the normalization of practices, such as engagement with the new practice, integration of the new practice in the organization’s overall goals, and structure and integration with existing working practices and skills [19-21]. Whether and how PHRs are adopted sustainably in health care practice is still unknown [14,22]. Therefore, the aim of our longitudinal study was to assess PHR adoption in general hospitals, academic hospitals, and private medical clinics using TARS over time (2021‐2023).

## Methods

### Study Design

This study belongs to a nationwide Dutch program called “VIPP 5” (Accelerated Program for Information Exchange between Patient and Professional 5) that ran from 2021 to 2023. The VIPP 5 program was an initiative by the Dutch Ministry of Health, Welfare and Sports by subsidizing health care organizations. It was aimed at promoting digital data exchange within specialized medical care and private medical clinics to improve digital communication both among health care providers and between providers and patients. VIPP 5 consisted of three core modules [23]: (1) Health care institutions were required to make digital health data available to patients through their PHR in accordance with the MedMij framework; (2) Organizations were expected to exchange digital health information with patients’ PHR, allowing patients to also send information back to the organization; and (3) Institutions were obliged to digitally exchange medical data with other health care providers. Patients were free to choose from various vendors to find a PHR that best suited their needs. The VIPP 5 program was administered and supported by the Dutch Hospital Association (NVZ), the Netherlands Federation of University Medical Centres (NFU), and Independent Clinics Netherlands (ZKN). The Dutch government offered financial incentives to the participating health care organizations (both hospitals and private medical clinics), aimed at accelerating PHR use. Our study explored PHR adoption with a longitudinal survey conducted in 2021, 2022, and 2023.

### Ethical Considerations

The data included no personal information from respondents. Participants did not receive any form of compensation for their contribution to this study. This study was approved by the Erasmus University Rotterdam ethical review board (ETH2021-0055) and respondents gave prior written informed consent.

### Population Selection

For this study, we aimed to include all contact persons of health care organizations participating in the VIPP 5 program. These contact persons (eg, project leaders and ICT employees) were the point of contact for PHR implementation within their organization. The participating healthcare organizations included hospitals, university medical centers, and private medical clinics across the Netherlands. These contact persons could represent their health care organization, as they were well positioned to identify relevant adoption barriers and facilitators. Participants were asked to report the name of their organization; no further personal information was collected as this was not necessary to answer the research question of this study.

### Study Procedure

On behalf of the Dutch Hospital Association, 1 author (CL-B) invited the VIPP 5 contact persons for the study by email. Besides information, the email included a link to a digital TARS questionnaire. The questionnaire was distributed at 3 points in time, in the autumn of 2021, 2022, and 2023. The questionnaires were open for 2 months per measurement. Participants were informed of the interim results to support the PHR adoption process. The number of invitations varied annually, due to new organizations entering or leaving the Dutch health care system, merges, or finishing VIPP 5. In total, the survey was sent to 291 participants in 2021, 302 in 2022, and 173 in 2023. The response rates were 49.1% (143/291 participants) in 2021, 43.4% (131/302 participants) in 2022, and 58.4% (101/173 participants) in 2023.

### Technology Adoption and Readiness Scale (TARS)

The TARS enables the evaluation of PHR adoption in organizations [19-21] by examining the implementation, embedment, and integration in different social contexts [19-21]. The original TARS contains 30 statements using a 6-point Likert scale for responses (1=completely disagree, 2=disagree, 3=disagree a little, 4=agree a little, 5=agree, and 6=completely agree) [19-21]. It includes 7 subscales: coherence (sense-making and understanding of the new practice), cognitive participation (engagement with the new practice), contextual integration (integrating the new practice in the organization’s overall goals and structure), skill set workability (integrating with existing working practices and skills), relational integration (the fit with existing professional relationships), interactional workability (whether the practice enables interactions), and reflexive monitoring (appraisal of the new practice) [19-21]. Table 1 presents an overview of the 7 subscales of TARS; Multimedia Appendix 1 presents the original TARS.

**Table 1.** Subscales of the Technology Adoption and Readiness Scale (TARS).

Subscale	Description
Coherence (2 items)	The process of sense-making and understanding that individuals and organizations have to go through in order to promote or inhibit the routine embedding of a practice to its users.
Cognitive participation (1 item)	The process that individuals and organizations have to go through in order to enroll individuals to engage with the new practice.
Contextual integration (10 items)	The degree to which the proposed eHealth system fits (or integrates) with the overall goals and structure of the organization (context), as well as the capacity of the organization to undertake the implementation.
Skill set workability (6 items)	The degree to which the eHealth initiative fits with existing working practices, skill sets, and perceived job role.
Relational integration (7 items)	The way in which professional groups relate to each other, and how well the proposed eHealth initiative fits (or integrates) with existing relationships, and the degree to which it promotes trust, accountability, and responsibility in intergroup relationships.
Interactional workability (5 items)	The degree to which the eHealth system enables (or impedes) the work of interactions between health professionals and patients, eg, a consultation.
Reflexive monitoring (1 item)	The informal and formal appraisal of a new practice once it is in use, in order to assess its advantages and disadvantages, and which develops users’ comprehension of the effects of a practice. These processes are energized by investments in appraisal made by participants.

This study used the Dutch translation of the validated TARS [21,24] (see Multimedia Appendix 2). This translation was performed by Wouters et al [24] following translation guidelines. Wouters et al [24] disaggregated 2 questions into 4 to enhance clarity, as originally, each contained 2 distinct elements. For instance, question 28 from the Coherence subscale—“The staff who work here have a shared understanding of what the system is for and how it is to be used”—was split into 2 distinct questions addressing staff and the organization, ensuring clearer interpretation by participants. Similarly, question 6 from the Contextual integration subscale—“This eHealth system is technically and organizationally compatible with other systems and agencies that we are required to work with”—was divided into questions about systems and (external) agencies to align with the Dutch health care context [21,24]. This resulted in 32 items in total instead of the original 30 items [21,24].

### Statistical Analysis

To assess the internal consistency and reliability of the TARS, we computed Cronbach ⍺. To estimate and test for differences, we conducted mixed-model analyses for each of the 7 subscales. We used mixed-model analysis. Mixed-model analysis can accommodate missing values [25] and is robust against violations of distributional assumptions [26]. First, we estimated and tested the intraclass correlations, based on the model with only the main effect of “time” as a factor. Next, we tested: (1) the model with main effects of 3 levels of time (2021, 2022, and 2023) and 3 levels of organization (academic hospital, general hospital, and private medical clinic), and (2) the model for the main effects of time and the interaction effect, assessing whether the development over time differed across the 3 types of organization. If the interaction effect was statistically significant (*α*=0.05), we computed simple effects of time for each type of organization separately; otherwise, we tested and interpreted the model (1) with only the main effects. Effect sizes were computed by dividing the differences between averages of two levels by average SD over time (ie, the square root of the average variance), which can be similarly interpreted as a Cohen *d* (small, medium, and large effects of 0.2, 0.5, and 0.8, respectively).

All tests were performed with IBM SPSS Statistics 27, with .05 significance level. Assuming 140 respondents with 3 observations and a correlation between repeated measures equal to 0.3, the statistical power is 0.8 to detect differences between time points: *f*^2^=.13 for effect size, which corresponds to a medium effect size (G*Power 3.1.9.7 [27]), and to detect differences between organizations for *f*^2^=.194, which corresponds to a medium-to-large effect [27].

### Content Analysis of Open-Text Explanations

The TARS consists of 32 statements using a 6-point Likert scale ranging from completely disagree to completely agree. Per TARS statement, participants were provided the opportunity to explain their answer (optional open explanation). These open-text explanations were analyzed using the content analysis method of Krippendorff [28]. This method encompasses five phases, namely (1) unitizing the data, (2) sampling the data, (3) recording and coding the data, (4) reducing the data, (5) inferring the data, and (6) narrating the results [28]. The first 4 phases focus on “data creation,” while the last 2 phases consider the interpretation and presentation of the results of the analysis [28]. During the first 2 phases, unitizing and sampling, all open explanations as filled in by the participants were collected and organized by subject in an overview, using the software tool Microsoft Excel. Next, during the third phase “Coding,” relevant responses to the study’s research question were selected. These 3 phases were performed by one author (DvdS) and checked by another author (AMW-J), both experienced in qualitative analysis. In the fourth phase “reducing data,” the relevant responses were summarized into short codes and sorted into themes, which were thoroughly discussed between 2 authors (DvdS and AMW-J) up to consensus. Next, we reflected on the data to infer its meaning and implications to refine the themes and determine thematic saturation of the found themes (phase 5, inferring). Finally, we described the results in a narrative format in the Results section (phase 6, narrating) [28].

## Results

### Descriptive Statistics

The TARS was completed by 143 of 291 (49.1%) participants in 2021, by 131 of 302 (43.4%) in 2022, and by 101 of 173 (58.4%) participants in 2023. All responses were included in this study. Per organization, responses varied throughout 2021 to 2023 ranging from 72% (n=65) to 91% (n=50) for private medical clinics, from 66% (n=65) to 87% (n=46) for general hospitals, and from 13% (n=1) to 83% (n=5) for academic hospitals (see Table 2 for an overview). In total, 55 organizations completed all 3 measurements during the 3 years (private medical clinics: n=33, general hospitals: n=22, and academic hospitals: n=0). In total, 72 organizations completed 2 of the 3 measurements (private medical clinics: n=30, general hospitals: n=38, and academic hospitals: n=4). Finally, in total, 66 organizations completed 1 of the 3 measurements (private medical clinics: n=30, general hospitals: n=33, and academic hospitals: n=3).

Table 3 provides an overview of descriptive statistics for responses to the TARS, for each organization type per year.

**Table 2.** Overview of the response rates per type of organization (private medical clinics, general hospitals, and academic hospitals).

Variables	Private medical clinics			General hospitals			Academic hospitals		
Year	2021	2022	2023	2021	2022	2023	2021	2022	2023
Total approached potential participants, n	101	90	55	87	98	53	8	8	6
Total responses (response rate), n (%)	74 (73)	65 (72)	50 (91)	64 (74)	65 (66)	46 (87)	5 (63)	1 (13)	5 (83)

**Table 3.** Results of the Technology Adoption and Readiness Scale (TARS) per subscale; mean (SD) per organization type per year.

Variables		Private medical clinics			General hospitals			Academic hospitals		
Year		2021	2022	2023	2021	2022	2023	2021	2022	2023
Responses, n		74	65	50	64	65	46	5	1^a^	5
Subscale, mean (SD)										
Coherence		3.68 (1.43)	3.89 (1.03)	4.20 (1.06)	2.78 (1.06)	3.24 (0.92)	3.67 (1.10)	3.10 (0.65)	3.00	3.20 (1.64)
Cognitive participation		4.41 (1.47)	4.38 (1.21)	4.76 (0.87)	3.64 (1.15)	3.79 (1.22)	4.11 (1.19)	3.60 (1.52)	5.00	4.80 (1.10)
Contextual integration		4.14 (0.78)	4.07 (0.51)	3.89 (0.68)	3.78 (0.56)	3.89 (0.68)	3.86 (0.58)	3.46 (0.84)	4.10	4.60 (0.32)
Skill set workability		3.68 (0.66)	3.82 (0.54)	3.71 (0.66)	3.58 (0.66)	3.50 (0.80)	3.69 (0.68)	3.50 (0.58)	4.00	4.48 (0.34)
Relational integration		4.33 (0.94)	4.11 (0.65)	4.15 (0.80)	4.14 (0.69)	3.93 (0.80)	4.24 (0.77)	3.94 (1.00)	4.14	4.81 (0.59)
Interactional workability		3.89 (1.17)	3.88 (0.71)	3.72 (0.88)	4.16 (0.61)	3.69 (0.87)	3.68 (0.87)	3.79 (0.60)	3.50	3.09 (1.62)
Reflexive monitoring		3.98 (1.36)	3.96 (0.98)	3.78 (1.19)	3.27 (1.14)	3.19 (1.27)	3.19 (1.60)	3.60 (1.52)	3.00	4.20 (1.64)

a As only 1 academic hospital responded to the questionnaire in 2022, SDs could not be computed.

Responses on the open questions came mostly from respondents in general hospitals and private medical clinics. Respondents in academic hospitals often did not give responses. In total, 404 responses were provided in 2021 (8.8% of 32 items × 143 participants=4576 potential open responses in total), 458 in 2022 (10.9% of 32 items × 131 participants=4192 potential open responses in total) and 356 in 2023 (11% of 32 items × 101 participants=3232 potential open responses in total). In total, participants provided 1218 responses to the open questions.

### TARS Reliability

The reliability of the Coherence subscale was considered acceptable to good (Cronbach ⍺=0.82 in 2021, 0.72 in 2022, and 0.85 in 2023) as was the reliability of relational integration (Cronbach ⍺=0.81 in 2021, 0.72 in 2022, and 0.70 in 2023) and interactional workability (Cronbach ⍺=0.89 in 2021, 0.73 in 2022, and 0.77 in 2023). The reliability of contextual integration ranges from questionable to acceptable (Cronbach ⍺=0.73 in 2021, 0.64 in 2022, and 0.67 in 2023), and while skill set workability shows questionable reliability (Cronbach ⍺=0.59 in 2021, 0.58 in 2022 and 0.55 in 2023), indicating lower internal consistency of the questions in these subscales. Cronbach ⍺ could not be calculated for cognitive participation or reflexive monitoring, as each subscale contained only 1 item.

### Mixed-Model Analysis Results

Intraclass correlations varied from 0.11 for interactional workability (*P*=.13) and 0.18 for reflexive monitoring (*P*=.04) to 0.28-0.42 for the other 5 scales (*P*<.001). This means that organizations generally differed systematically across years on the subscales. Consequently, the data were statistically dependent and should therefore be analyzed with mixed models. Table 4 below shows the results of the mixed-model analyses. As an interaction effect was found only for contextual integration, we interpreted the main effects for the other 6 subscales.

**Table 4.** Results of the Technology Adoption and Readiness Scale (TARS) mixed-model analysis over time (2021, 2022, and 2023) and per organization (private medical clinic, general hospital, and academic hospital) and the interaction effect.

Subscale		Intraclass correlations (ICC)		Interaction effect		Effect of organization type^a^		Effect of time		Effect size (β/SE)^b^^,^^c^	
		ICC	*P* value	*F* test (*df*)	*P* value	*F* test (*df*)	*P* value	*F* test (*df*)	*P* value	2022‐2023	2021‐2023
Coherence		0.39	<.001	0.70 (2)	.59	16.21 (2)	<.001	10.67 (2)	<.001	0.24	0.58
Cognitive participation		0.42	<.001	0.95 (2)	.44	11.2 (2)	<.001	4.50 (2)	.01	0.20	0.33
Contextual integration		0.35	<.001	5.42 (2)	<.001	3.411 (2)	.04	4.54 (2)	.01	−0.33^d^	−0.38^e^
Skill set workability		0.28	<.001	1.65 (2)	.17	3.12 (2)	.047	4.24 (2)	.02	0.21	0.34
Relational integration		0.39	<.001	1.99 (2)	.10	1.26 (2)	.29	2.92 (2)	.06	0.24	0.06
Interactional workability		0.11	.13	1.22 (2)	.31	0.96 (2)	.39	4.95 (2)	.01	−0.08	−0.41
Reflexive monitoring		0.18	.042	0.34 (2)	.85	11.11 (2)	<.001	.26 (2)	.77	−0.09	−0.10

a The statistical results of “time” and “organization type” are for the model with only the 2 effects, excluding the interaction effect.

b β here is the unstandardized regression coefficient.

c The difference in the dependent variable between 2023 and 2021 or between 2023 and 2022, divided by the square root of the average variance at each time point (2021, 2022, and 2023).

d Reported −0.33 (private medical clinics), 0.17 (general hospitals), and 0.87 (academic hospitals).

e Reported for −0.38 (private medical clinics), 0.28 (general hospitals), and 1.21 (academic hospitals).

The development of contextual integration differed over time between the 3 types of organizations (*F*_4,137_=5.417; *P*=<.001). In private medical clinics, it decreased from 2021 to 2023 (Cohen *d*_1_=−0.38) and from 2022 to 2023 (Cohen* d*_2_*=*−0.33), whereas it increased in general hospitals (Cohen* d*_1_=0.28 and Cohen* d*_2_=0.17) and in academic hospitals (Cohen* d*_1_=1.21 and Cohen *d*_2_=0.87).

Improvements over time were observed for coherence, indicating that sense-making and understanding to embed PHR in health care practice increased over time (*P*<.001; *d_1_*=0.58 and *d_2_*=0.24). Cognitive participation increased as well, indicating that the engagement of individuals increased over time (*P*=.01; *d_1_*=0.33 and *d_2_*=0.20). Furthermore, skill set workability improved, indicating improved fit of PHRs with existing working practices, skill sets, and perceived job roles (*P*=.02; *d_1_*=0.34 and *d_2_*=0.21). A decrease over time was observed for interactional workability, indicating that PHRs were considered less suitable to improve interactions between health care providers and patients in all types of organizations (*P*=.01; *d_1_*=−0.41 and *d_2_*=−0.08). No changes were observed for relational integration, indicating that the integration of the PHR in professional relationships and trust, accountability, and responsibility in intergroup relationships remained stable over time (*P*=.06; *d_1_*=0.06 and *d_2_*=0.24). Finally, no changes were observed for reflexive monitoring, indicating that assessment of PHR advantages and disadvantages and the development of users’ comprehension of the effects of a practice remained stable over time (*P*=.77; *d_1_*=−0.10 and *d_2_*=−0.09).

Concerning the effects of organization type, organizations differed on the following scales, with general hospitals consistently scoring lower than private medical clinics (results of *t* tests comparing both organizations) on coherence (*t*_178_=−5.52; *P*<.001), cognitive participation (*t*_181_*=*−4.73; *P*<.001), and reflexive monitoring (*t*_152_=−4.68; *P*<.001). Organizations did not differ on skill set workability (*t*_186_=−1.64; *P*=.1), relational integration (*t*_181_=−1.44; *P*=.15), and interactional workability (*t*=.12; *P*=.91).

### Results of the Qualitative Open-Text Explanations

#### Coherence

Coherence is the sense-making and understanding needed to embed a practice. In 2021, when PHRs were not yet widely implemented, respondents lacked an understanding and awareness of PHRs’ possibilities, and some expressed skepticism about their added value. By 2022, respondents reported a clearer grasp of the purpose, yet the practical implications remained ambiguous due to the limited availability of PHRs. By 2023, although PHRs were generally functional, health care organizations encountered technical difficulties, particularly when integrating data from EHRs in PHRs. Over the years, respondents from private medical clinics indicated more often that they had a clear understanding of the purpose and added value of PHRs, while these aspects often remained unclear for respondents from general hospitals.

The purpose of the PHR is clear, but not everyone in the organization is fully aware of it yet […]. How it should be used in practice is not entirely clear to me, as it has not been completely implemented. This process will likely become clearer in the coming year.

[R12-PMC2022-Coherence]

#### Cognitive Participation

Cognitive participation is the process to engage individuals with new practices. Respondents indicated that due to the delayed implementation of PHRs, staff engagement was minimal in 2021. In 2022, general hospital respondents doubted the added value of PHRs, which may have been caused by a lack of involvement of potential users in the development and implementation phases. In 2023, most respondents noted communications efforts made to enhance professionals’ understanding of the PHR. With increased insights into the purpose of PHRs, respondents indicated that health care professionals felt more committed to offer PHRs to patients. Despite their commitment, they noted that the technical integration of available data from EHRs within PHRs remained problematic, rendering the use of PHRs cumbersome. Throughout the 3 years, respondents from private medical clinics consistently indicated commitment, while this varied in general hospitals.

Although the PHRs are not sufficiently suitable for the patients yet, the management team is fully committed.

[R1-AH2023-Cognitive participation]

We wonder if our target audience, […], is able to use it and can understand its use.

[R185-PMC2023-Cognitive participation]

#### Contextual Integration

Contextual integration is the degree to which the technology integrates with the organizations’ goals, structures, and capacity. In 2021, respondents indicated that the PHRs’ impact on workflows could not be assessed, and therefore it was unclear whether the benefits would outweigh the costs. While some organizations had adequate funds for long-term implementation, others faced insufficient long-term financing to integrate the preferred PHR functionalities or they feared financial penalties for unmet project targets. Private medical clinics and general hospitals indicated a need for more time to implement and especially to embed PHRs in work processes and services, and general hospitals also indicated a lack of dedicated personnel to implement PHRs. By 2022, respondents had problems supporting PHR use by patients because PHRs are managed externally. Respondents ascribed the lack of adoption to patients’ unawareness of PHR’s existence. By 2023, some respondents indicated that the PHR required workflow changes, for instance, in entering patient data. Some mentioned the complexity of aligning different systems and data from different organizations, which hindered data exchange because external partners are not always linked to PHRs, thus limiting the amount of information available to patients. According to respondents in academic hospitals, the PHRs did not adequately meet patient needs, mainly due to technical barriers for users, such as difficult logins for patients.

Many of our external partners are not linked to a PHR yet, or are unable to exchange data with other healthcare providers.

[R64-H2023-Contextual integration]

A patient has to take too many steps to log in to the PHR compared to logging in to the hospital patient portal. […] Half of the log-in attempts go wrong, and patients do not understand why. Moreover, the patient has to go through all the steps every time. We often hear [complaints about] this from users.

[R81-PMC2023-Contextual integration]

#### Skill Set Workability

Skill set workability is the technology fit with existing working practices, skill sets, and perceived job roles. In 2021 and 2022, respondents were uncertain about the impact of PHRs on professional autonomy, workload, collaboration, or employee skills. In 2022 and 2023, some respondents indicated that while initial PHR implementation would be time-consuming, they believed it would eventually reduce the professional workload. By 2023, perceptions of the level of professionals’ digital skills varied, suggesting that some may need training to be able to adequately support patients. Some organizations still faced uncertainties about the required skills and associated workload. Some professionals perceived the PHR as obligatory, resulting in a feeling of diminished autonomy.

In the beginning [implementation] will take more effort and it will be harder to handle. When practice and processes are embedded, the workload will go down. But workload will go up if the PHR is not sufficiently stable or fails.

[R67-H2022-Skill set workability]

The PHR partly gives me a feeling of autonomy in my work. However, technology should not get the upper hand. Healthcare is people work after all. It does offer opportunities to innovate and improve care but the legislation and regulations must be in line with (limiting the administrative burden).

[R35-PMC2023-Skill set workability]

#### Relational Integration

Relational integration is the integration of the technology in professional relationships and trust, accountability, and responsibility in intergroup relationships. From 2021 to 2023, respondents consistently expressed concerns regarding the privacy and security of patient data in PHRs. They highlighted a lack of control over numerous PHRs that might exploit patient data commercially. The lack of transparency, combined with the potential for patients to unknowingly share sensitive information, led to calls for a single trusted entity to oversee PHR vendors and ensure data security. By 2023, while some respondents trusted PHRs because of government regulations, others remained concerned about the commercial nature and potential liability issues for healthcare organizations.

I find the fact that there are so many different PHRs risky. I cannot imagine that they all offer good secure access and all have the patient’s best interests at heart. There is too much money in this market for that. Patients often don't know what they are choosing. They give up too much privacy-sensitive information.

[R4-PMC2021-Relational integration]

#### Interactional Workability

Interactional workability is the degree to which the technology enables the work of interactions between health professionals and patients. Some respondents noted that the technical challenges made it significantly difficult to integrate PHRs into work routines with patients. They worried that patients would be overwhelmed by excessive information, especially lab results without proper medical context. In 2023, most respondents were still uncertain whether PHRs genuinely improved patient-professional interaction. Some viewed it as time-consuming, while others believed it enhanced the quality of consultations. Respondents from private medical clinics hoped for positive outcomes, while those from both general and academic hospitals felt it was too early to determine.

It’s too early to decide [if the technology has positive effects for clients], but being able to monitor at home and the transparency are good [for patients].

[R33-H2023-Interactional workability]

You can’t do much in consultation of ten minutes on average. Consultation quality did become better, more specific. […] The innovation mainly affects indirect patient time, mainly consultation preparation and admission planning. It has little effect on direct patient time [for physicians]. So there’s little causality between the innovation and quality interaction.

[R2-AH2023-Interactional workability]

#### Reflexive Monitoring

Reflexive monitoring is the appraisal of new practices to assess its advantages and disadvantages and develop users’ comprehension of the effects of a practice. All respondents indicated that monitoring and evaluating PHRs did not apply in 2021 and 2022. By 2023, academic hospitals had evaluated PHRs and found their usage to be effective. However, concerns were raised about the hospital’s limited influence over PHR vendors, leading to uncertainty about future developments. General hospitals and private medical clinics did not perform evaluation, and no differences in their open explanations could be found.

We have little say on further developments. That applies both to the providers and the functionality of electronic health records.

[R33-H2023-Reflexive monitoring]

## Discussion

### Principal Findings

This study provides insight into the adoption of PHRs across private medical clinics, general hospitals, and academic hospitals from 2021 to 2023. We found improvements in several areas of technology adoption. Coherence, sense-making, and understanding of PHRs for embedment in health care practice increased over time, as did cognitive participation, showing increased engagement and commitment of individuals to PHRs, which according to the respondents, was due to improved communication about PHRs. Better skill set workability indicated an improved fit of PHRs with existing working practices, skills, and perceived job roles.

However, we found some barriers to the adoption of PHRs. While contextual integration of PHRs with organizations’ goals, structures, and capacity improved in general and academic hospitals, this decreased in private medical clinics. This difference was explained in open responses by variation in available funding and financial targets across organizations. Limited adoption by patients and health care professionals, technical barriers, and uncertainties about the required skills and concerns about associated workload were also mentioned. Interactional workability was found to decrease over time, indicating that the PHR was considered less suitable to improve interactions between health care providers and patients. Technical challenges would require significant effort to integrate PHRs into work routines with patients, and the technical integration of EHRs with PHRs remained problematic. Some health care professionals remained uncertain whether PHRs genuinely improved patient-professional interaction.

While relational integration in professional relationships remained stable over time, a lack of control and transparency regarding PHRs led to privacy and security concerns. For reflexive monitoring, academic hospitals evaluated PHRs and found their usage to be effective. General hospitals scored consistently lower than private medical clinics on coherence, cognitive participation, and reflexive monitoring. This was confirmed in the open responses, except for reflexive monitoring, as in this case, the open responses were similar and we could not identify any difference between organizations.

### Strengths and Limitations

A first strength of this study is the longitudinal assessment of PHR adoption, which is understudied in previous literature; we collected data throughout 3 years. A second strength is that relevant domains for the adoption were evaluated with a mixed-methods approach, both qualitative (open-text) and quantitative responses were collected using the validated (extended) Dutch TARS. The third strength is that this study followed PHR monitoring of the adoption and implementation from an early stage, from the start of the implementation, onwards. Our results can be used as input to improve PHRs and their sustainable implementation [16].

A limitation of the study is that we used the original TARS instead of the updated version of the TARS; the NOMAD questionnaire [29,30]. The original TARS was selected because the Dutch translation of the NOMAD had not been validated in Dutch at the start of our study. However, both questionnaires were derived from the NPT [19-21], which is used to evaluate individual and organizational factors that influence the adoption process, and both questionnaires are validated. A second limitation is answering the open-text questions for each statement of the TARS was optional, not obligatory. Therefore, only some respondents (8.8% in 2021, 10.9% in 2022, and 11% in 2023) explained their ratings. Because of the reliance on these self-reported data, bias may occur, for instance, by participants responding in a socially desirable way or misunderstanding questions [31]. Furthermore, individuals who are particularly concerned or dissatisfied are more likely to provide detailed feedback [32-34]. However, our research design in which we used statements (quantitative) as well as room for open explanation (qualitative) enhanced the reliability and validity of the data; we could identify whether participants understood the question, and we think the participants answered in an honest way as they mostly provided detailed feedback on PHRs. A third limitation of the statistical analysis is the unequal distribution of responses across organizational types. However, the unequal distribution reflects the population distribution. For instance, the Dutch health care system only has 7 academic hospitals, of which we included 5 in our sample. In addition, the number of responses varied across years and organization types, as not all organizations participated in each measurement (2021, 2022, and 2023). This was due to new organizations entering or leaving the Dutch health care system, participating in organizational merges, or finishing VIPP 5. Consequently, the number of contact persons of VIPP 5 varied throughout the years. To address this, we conducted mixed-model analysis, which effectively deals with missing data. Both the unequal sample sizes and the missing data do not pose a threat to the mixed-model analyses that we performed. The sample size of this study (143 at the first measurement, 131 at the second measurement, and 101 at the third measurement) was generally lower than the planned 140. Consequently, our statistical power to detect the assumed effect sizes in our power analyses was a bit lower than 0.8 (but still larger than 0.7, assuming a sample size of n=125 at all measurements). Furthermore, this study included representatives (contact persons) rather than end users (patients) or health care professionals, which would have enhanced the representativeness of the findings. However, as the study aim was on PHR adoption, we believe project leaders offered a more comprehensive perspective on adoption and normalization processes. Finally, as the study was conducted only in the Netherlands in the Dutch health care context, the generalizability to other countries may be limited.

### Comparison With Previous Work

Our study found that PHRs were adopted in health care practice and work routines only to a limited extent, mainly due to the limited adoption by patients. Respondents indicated this is an interplay; if patients do not use PHRs, health care professionals will not use or recommend it either and vice versa. In line with previous research [14,35], our study found that implementing PHRs is challenging in terms of adoption by patients [14], and that it is often complicated to change communication patterns of patients [35]. Our study indicated that both patients and health care professionals lacked awareness of the existence and potential of PHRs. This is in line with a review by Papadoulos et al [36], highlighting the public’s unawareness of EHRs, what these entail and their purpose. This may need to be improved to increase the uptake of PHRs. Several of our respondents indicated that they doubt the added value of a PHR for patients in addition to a regular patient portal. This finding resembles a study by Crameri et al [10], which reports that patients, including potential users, may not feel a need to interact with their health information in PHRs. In contrast, a cohort study by the Dutch Patient Federation on the use of Dutch PHRs indicated that two thirds of patients perceive the Dutch PHR as useful and intend to use it in the future [37], which indicates a potential increase of PHR users in the future.

Our study revealed that technical barriers, such as patient portal login difficulties, are a reason for the limited adoption by patients. The role of accessibility in PHR adoption is also found in previous studies [37,38]. Perotti et al [39] evaluated an e-learning to promote PHR competence and found how e-learning enhanced knowledge of PHRs, skill acquisition to use PHRs, and improved task completion. PHR providers are encouraged to develop and provide a publicly accessible e-learning for PHR users [39].

A cohort study by Doeven [37] showed that patient-users perceive the Dutch PHR as reliable [37] mainly because of 2-factor authentication and the use of a personal digital ID that every Dutch citizen can obtain from the government. In contrast, our respondents expressed concerns about the reliability, privacy, security, and safety of PHRs and attributed this to the commercial interests of different vendors. These findings were in line with the results of a scoping review performed by Papadopoulos et al [36] on public trust in EHRs. Similar to this study, privacy was identified as a primary concern in this review because of the concern that third parties may commercially exploit persons’ health data, with the fear of hackers, unauthorized access, selling information for profit, or identity fraud. In line with Papadopoulos et al [36], we found that assurance of privacy, security, and safety of data would be crucial to improve trust in PHRs. In line with the cohort study by Doeven [37], Papadopoulos et al [36], as well as a review by Fennelly et al [40], we also found that security and privacy measures such as 2-factor authentication would increase the sense of security of PHRs. By focusing on issues related to PHRs security and privacy in future research, more insights will be gained on appropriate measures, as recommended in the review by Roehrs et al [5].

Another finding of our study is the relevance of the technical integration of available data from EHRs within PHRs. A scoping review of Fennelly et al [40] also identified technical interoperability between systems and data as an obstacle. Fennelly et al [40] suggested using internationally recognized open standards supporting homogenous functional models, a centralized data repository that requires organizing data into defined standards.

### Implications for Practice

First, to improve usability for patients, PHR accessibility should be enhanced, for instance, by simplifying the log-in procedure. By providing a publicly accessible e-learning for PHR users, their knowledge, task completion, and skill acquisition could be enhanced [39]. In line with recommendations for eHealth implementation [41], the alignment of data in different structures in PHRs should be improved. To achieve this, health care organizations should collect and store patient data in standard ways, by following internationally recognized open standards supporting homogenous functional models, a central data repository, and organizing data into standardized and common formats by using standardized terminologies [40].

Second, to enhance PHR adoption by patients and health care professionals, it is important to enhance their awareness of the existence and potential of PHRs. If health care professionals are adequately informed, they could recommend their use to patients as well. To reach patients and increase PHR adoption, health care organizations could use public media campaigns and internal communication means to explain the relevance of PHRs, in line with recommended organizational strategies for promoting patient and provider uptake of PHRs [42]. Strategies for promoting acceptance of PHRs emphasize addressing organizational cultures, redesigning work processes, training professionals, and providing technical support and monitoring [42].

Third, as respondents expressed the need for a single trusted entity to oversee PHR vendors to ensure data security, the awareness of security and privacy guidelines, and assessment of these by a National trustworthy organization is needed. Security measures such as the 2-factor authentication to enhance privacy and security are suggested in other studies [41].

### Recommendations for Future Research

Future research on PHR use by patients and health care professionals should be continually and iteratively evaluated to determine whether PHRs actually adhere to users’ needs. PHRs should be improved on an ongoing basis to ensure and enhance the added value, usability, and accessibility [16]. Future research should also focus on continuously and iteratively evaluating PHRs’ security, privacy, and trust. Many respondents indicated that PHRs were still being implemented during our study. Future research could further explore PHR adoption in the coming years, incorporating more insights into both organizational and national contexts too.

### Conclusions

From 2021 to 2023, PHR adoption improved in general hospitals, academic hospitals, and private medical clinics in the Netherlands within several domains of PHR adoption. However, several barriers remained, including technical barriers, financial constraints, and privacy and security concerns. To overcome these barriers, the technical integration of available data from the EHR within the PHR should be improved and privacy-related issues should be resolved. Consistent communication about the potential of PHRs is required to increase the awareness that will enhance both PHR engagement and adoption by the target groups, patients, and health care professionals.

## Supplementary material

10.2196/71915 Multimedia Appendix 1 The original Technology Adoption and Readiness Scale (TARS).

10.2196/71915 Multimedia Appendix 2 The Dutch translation of the Technology Adoption and Readiness Scale (TARS).

## Abbreviations

EHR: electronic health record
NPT: Normalization Process Theory
PHE: personal health environment
PHR: personal health record
PP: patient portal
TARS: Technology Adoption and Readiness Scale
VIPP 5: Accelerated Program for Information Exchange between Patient and Professional 5
