# Supplementary material for: Adoption of Personal Health Records in Dutch Hospitals and Private Medical Clinics: Longitudinal Study
Source: J Med Internet Res. 2025 Aug 11;27:e71915. doi: 10.2196/71915 (PMC12501903; doi:10.2196/71915)
Supplement: Multimedia Appendix 1 [file jmir-v27-e71915-s001.docx]

Multimedia Appendix 1. The Original Technology Adoption and Readiness Scale (TARS) [21]

The original TARS contains 30 statements using a 6-point Likert scale for responses (1=completely disagree, 2=disagree, 3=disagree a little, 4=agree a little, 5=agree, 6=completely agree) [21].

1. Contextual integration – The eHealth system is adequately resourced financially

2. Contextual integration – Sufficient organizational effort has gone into supporting the eHealth system

3. Contextual integration – The eHealth system is a different way of working

4. Contextual integration – The rewards of using the eHealth system outweighs the effort

5. Contextual integration – Government policy initiatives are supportive of this eHealth system

6. Contextual integration – This eHealth system is technically and organizationally compatible with other systems and agencies that we are required to work with

7. Contextual integration – This eHealth system fits in with the priorities and challenges of our organization

8. Contextual integration – This organization has a culture that is supportive of change

9. Contextual integration – There is a culture in this organization of involving staff in planning and development

10. Skill set workability – Using the eHealth system makes me feel autonomous in my work

11. Skill set workability – Using the eHealth system requires co-operation with other staff

12. Skill set workability – The workload involved in using the eHealth system is manageable

13. Skill set workability – In using the eHealth system, the allocation of work between individuals is appropriate

14. Skill set workability – The skills I have are appropriate for using the eHealth system

15. Skill set workability – The skills needed to use the eHealth system are easily learned

16. Relational integration – I have confidence that using the eHealth system does not put patients at risk

17. Relational integration – Using the eHealth system is an efficient use of time

18. Relational integration – In using the eHealth system, responsibilities are divided between individuals appropriately

19. Relational integration – In using the eHealth system, I understand my accountability for my work

20. Relational integration – In using the eHealth system, I understand my liability for my practice

21. Relational integration – Technical back-up in using the eHealth system is available if I need it

22. Relational integration – I believe there is good evidence about the clinical effectiveness of using the eHealth system

23. Interactional workability – There is some flexibility in how the eHealth system can be used

24. Interactional workability – Using the eHealth system leads to positive outcomes for patients

25. Interactional workability – Using the eHealth system involves the right amount of time spent with patients (on the telephone)

26. Interactional workability – In using the eHealth system, the quality of professional and patient interaction is good

27. Interactional workability – The eHealth system is easy to use

28. Coherence – The staff who work here have a shared understanding of what the system is for and how it is to be used^a^

29. Cognitive participation – The staff here are committed to making the system work

30. Reflexive monitoring – There are ongoing mechanisms for monitoring and appraising how this eHealth system is used
