# Supplementary material for: Adoption of Personal Health Records in Dutch Hospitals and Private Medical Clinics: Longitudinal Study
Source: J Med Internet Res. 2025 Aug 11;27:e71915. doi: 10.2196/71915 (PMC12501903; doi:10.2196/71915)
Supplement: Multimedia Appendix 2 [file jmir-v27-e71915-s002.docx]

Multimedia Appendix 2. The Dutch Translation of the Technology Adoption and Readiness Scale (TARS) [21, 24]

**Gedeelde betekenis *(Coherence)* (1 vraag in originele versie, 2 vragen in Nederlandse vertaling)**

GB_T.1 Bij de professionals is er een eenduidig beeld over waar [de technologische innovatie] voor dient en hoe het gebruikt moet worden.

GB_T.2 Bij de organisatie is er een eenduidig beeld over waar [de technologische innovatie] voor dient en hoe het gebruikt moet worden.

**Meedenken *(Cognitive participation)* (1 vraag)**

M_T.3 Binnen de organisatie zetten alle betrokkenen zich in om van [de technologische innovatie] een succes te maken.

**Context *(Contextual integration)* (9 vragen in originele versie, 10 vragen in Nederlandse versie)**

C_T.4 Er zijn voldoende financiële middelen beschikbaar voor [de technologische innovatie].

C_T.5 Er zijn voldoende organisatorische inspanningen verricht om [de technologische innovatie] te ondersteunen.

C_T.6 [de technologische innovatie] houdt een andere manier van werken in.

C_T.7 De baten van het gebruik van [de technologische innovatie] wegen op tegen de inspanningen.

C_T.8 De overheid stimuleert toepassingen zoals [de technologische innovatie].

C_T.9 [de technologische innovatie] sluit, wat betreft techniek en organisatie, aan bij de andere systemen waarmee de organisatie werkt.

C_T.10 [de technologische innovatie] sluit aan bij onze samenwerking met externe partners.

C_T.11 [de technologische innovatie] past binnen de prioriteiten en uitdagingen van onze organisatie.

C_T.12 Deze organisatie heeft een cultuur die veranderingen steunt.

C_T.13 Deze organisatie heeft een cultuur om medewerkers te betrekken bij strategie en ontwikkeling.

**Vaardigheden *(Skill set workability)* (6 vragen)**
V_T.14 Het gebruik van [de technologische innovatie] geeft mij een gevoel van autonomie in mijn werk.

V_T.15 Het gebruik van [de technologische innovatie] vereist samenwerking met andere medewerkers.

V_T.16 De werklast die het gebruik van het [de technologische innovatie] met zich meebrengt is te hanteren.

V_T.17 Bij het gebruik van [de technologische innovatie] is de werkverdeling tussen mij en mijn collega’s goed.

V_T.18 Ik bezit de benodigde vaardigheden om [de technologische innovatie] te kunnen gebruiken.

V_T.19 De vaardigheden die nodig zijn om [de technologische innovatie] te gebruiken zijn eenvoudig te leren.

**Werkproces *(Relational integration)* (7 vragen)**

W_T.20 Ik heb er vertrouwen in dat het gebruik van [de technologische innovatie] niet riskant is voor de patiënten/cliënten.

W_T.21 Het is, wat betreft tijd, efficiënt om [de technologische innovatie] te gebruiken.

W_T.22 Bij het gebruik van [de technologische innovatie] zijn de verantwoordelijkheden naar behoren verdeeld tussen de individuele medewerkers.

W_T.23 Ik begrijp waarop ik verantwoordelijkheid moet afleggen wanneer ik werk met het [de technologische innovatie].

W_T.24 Ik begrijp waarvoor ik beroepsmatig aansprakelijk kan worden gesteld wanneer ik gebruik maak van [de technologische innovatie].

W_T.25 Er is technische ondersteuning bij het gebruik van [de technologische innovatie] beschikbaar wanneer ik die nodig heb.

W_T.26 Ik geloof dat er voldoende bewijs is voor de effectiviteit van [de technologische innovatie].

**Menselijke interactie *(Interactional workability)* (6 vragen)**

MI_T.27 Er zit enige flexibiliteit in de manier waarop [de technologische innovatie] kan worden gebruikt.

MI_T.28 Het gebruik van [de technologische innovatie] leidt tot positieve effecten bij de cliënten.

MI_T.29 Door het gebruik van [de technologische innovatie] kan ik de juiste hoeveelheid tijd met de cliënten doorbrengen.

MI_T.30 Bij het gebruik van [de technologische innovatie] is de kwaliteit van de interactie tussen zorgprofessional en cliënt goed.

MI_T.31 [de technologische innovatie] is gemakkelijk te gebruiken.

TL_T.32 De wijze waarop [de technologische innovatie] wordt gebruikt, wordt systematisch gemonitord en geëvalueerd.

^a^The Dutch translation of the TARS contains 32 statements using a 6-point Likert scale for responses (1=Helemaal mee oneens, 2=Mee oneens, 3=Beetje mee oneens, 4=Beetje mee eens, 5=Mee eens, 6=Helemaal mee eens) [21, 24].

^b^In this Dutch translation of the TARS, two questions were disaggregated into four distinct questions to better capture their dual aspects, resulting in 32 items in total instead of the original 30 items [21, 24]. Specifically, question 28 from the Coherence subscale—“The staff who work here have a shared understanding of what the system is for and how it is to be used”—was turned into separate questions for staff, and for the organization as a whole. Similarly, question 6 from the Contextual integration subscale—“This eHealth system is technically and organizationally compatible with other systems and agencies that we are required to work with”—was divided into questions about systems and (external) agencies to align with the Dutch healthcare context [21, 24].
